# Complete genome sequences of *Trueperella abortisuis* isolated from porcine abortion cases in Kumamoto, Japan

**DOI:** 10.1128/mra.00862-26

**Published:** 2026-10-06

**Authors:** Sayaka Nishikawa, Yoshino Mizuno, Mariko Okamoto, Takashi Mada, Daisuke Takamatsu, Momoko Nakayama, Akari Tachibana, Masahiro Eguchi, Keiji Nakamura

**Affiliations:** 1 Department of Bacterial Diseases, National Institute of Animal Health, National Agriculture and Food Research Organization, Ibaraki, Japan; 2 Kumamoto Meat Inspection Office, Kumamoto, Japan; 3 Graduate School of Medical Sciences, Kyushu University 12923, Fukuoka, Japan; University of Manitoba, Winnipeg, Manitoba, Canada

**Keywords:** *Trueperella abortisuis*, porcine abortion, complete genome

## Abstract

*Trueperella abortisuis* is an emerging pathogen associated with porcine abortion; however, its genomic information remains limited. To expand the genomic resources of *T. abortisuis*, we report the complete genome sequences of four strains isolated from the lungs of aborted pig fetuses in Kumamoto, Japan, in 2020.

## ANNOUNCEMENT

*Trueperella abortisuis* is a gram-positive, diphtheroid-shaped bacterium first isolated from an aborted sow in Japan in 2009 (1) and has since been associated with porcine abortion in several countries (2–4). To date, only two genome sequences of *T. abortisuis* are publicly available, originating from the placenta of an aborted pig in Japan and the uterus of an aborted goat in Canada (5). While this bacterium has frequently been associated with pulmonary lesions in aborted fetuses (3, 6), no genome sequences of lung-derived isolates have been reported. We, therefore, performed complete genome sequencing of four lung-derived isolates.

In 2020, three cases of porcine abortion occurred on a farm in Kumamoto, Japan. The placenta and five fetuses from one of the affected sows were investigated to identify the causative agent. After surface disinfection, the placenta and fetal lungs were cut, and their cross-sections were stamped directly onto 5% sheep blood agar plates (Nissui Pharmaceutical), followed by streaking with an inoculation loop. Incubation at 37°C under 10% CO_2_ for 2 days yielded only common bacteria from the placenta and one lung, whereas unique bacterial colonies were isolated from the remaining four fetal lungs. These isolates were α-hemolytic, gram-positive, short rod-shaped bacteria and were identified as *T. abortisuis* based on 16S rRNA gene sequencing and BLAST analysis. Their association with the abortion cases was supported by histopathological examination, which revealed necrotizing placentitis with neutrophil infiltration and bacterial aggregates, as well as suppurative pneumonia in some fetuses. In addition, immunohistochemical analysis for *Toxoplasma gondii* antigen was negative, further supporting *T. abortisuis* as the most likely causative agent of the abortion.

Genomic DNA was extracted using the DNeasy Blood & Tissue Kit (QIAGEN), followed by RNA exclusion using RNase Cocktail Enzyme Mix (Invitrogen), purification with AMPure XP beads (Beckman Coulter), and quantification using the Qubit dsDNA HS assay kit (ThermoFisher Scientific).

Hybrid sequencing (Illumina and Oxford Nanopore) was employed to obtain high-accuracy complete genomes. Illumina libraries were prepared using the Nextera DNA Prep kit and sequenced on an iSeq 100 (both Illumina) to generate 150-bp paired-end reads. Nanopore libraries were prepared using the Rapid Sequencing Kit 96 V14 (SQK-RBK114.96; Oxford Nanopore Technologies [ONT]) and sequenced on a MinION device with an R10.4.1 flow cell (ONT). Raw Nanopore reads were base-called using Dorado v1.0.0 in the super-accurate (SUP) mode (model: dna_r10.4.1_e8.2_400bps_sup@v5.2.0) (7). These reads were then assembled using Hybracter v0.11.0 (8) in the hybrid mode, with Flye (9)-determined replicon circularity. To further specify the species, taxonomic assignment was performed using GTDB-Tk v2.4.0 (10), and assembly completeness was assessed using BUSCO v5.8.3 (11) with the *Actinomycetaceae* lineage data set. All reagents and kits were used according to the manufacturer’s instructions. All bioinformatic tools were run with default parameters.

All four isolates were confirmed as *T. abortisuis*. The assembled genomes showed all 98.6% completeness and nearly identical GC contents, ranging from 64.17% to 64.19% (Table 1). However, variation in chromosome size was observed among the strains (2,455,793–2,463,136 bp), suggesting genetic heterogeneity. No plasmids were detected in any of the four genomes.

**TABLE 1:** General features, assembly metrics, and accession numbers of four *T. abortisuis* genome sequences

Strain	Genome size (bp)	GC content (%)	Completeness/contamination (%)^*a*^	Coverage (x)^*b*^	GenBank accession no.^*c*^	Nanopore reads			Illumina reads		
						Total no. of bases (bp)	No. of reads	SRA accession no.	Total no. of bases (bp)	No. of reads	SRA accession no.
DTK483	2,463,136	64.17	98.6/0.00	95	AP050802	360,300,308	351,702	DRR959495	170,034,759	1,161,090	DRR959491
DTK484	2,455,793	64.19	98.6/0.00	84	AP050803	431,017,708	105,940	DRR959496	155,304,092	1,050,750	DRR959492
DTK485	2,459,362	64.18	98.6/0.00	87	AP050804	1,487,432,330	559,960	DRR959497	168,586,518	1,143,976	DRR959493
DTK486	2,456,415	64.19	98.6/0.00	96	AP050805	635,181,598	512,242	DRR959498	160,043,695	1,089,784	DRR959494

^*a*^ Completeness (BUSCO, as described in the main text) and contamination (CheckM v1.2.2 [12]), lineage-specific workflow; phylum *Actinobacteria* (now *Actinomycetota*) marker set, UID1454; 201 marker genes were assessed independently.

^*b*^ Nanopore reads were subsampled to approx. 100× coverage using Filtlong in Hybracter prior to assembly to ensure optimal assembly performance.

^*c*^ Genomes were annotated using DFAST (13) prior to submission.

## Data Availability

The complete genome sequences of the isolates (DTK483–DTK486) have been deposited in DNA Data Bank of Japan (DDBJ) under BioProject accession number PRJDB40816 and BioSample accession numbers SAMD01840682 to SAMD01840685. The raw sequence data have been deposited in the DDBJ DRA under accession numbers DRR959491 to DRR959498.
